# Electrolyzed Hypochlorous Acid (HOCl) Aqueous Solution as Low-Impact and Eco-Friendly Agent for Floor Cleaning and Sanitation

**DOI:** 10.3390/ijerph20186712

**Published:** 2023-09-05

**Authors:** Alessandro Gessi, Paolo Formaglio, Bruno Semeraro, Daniela Summa, Elena Tamisari, Elena Tamburini

**Affiliations:** 1 ENEA Research Center, SSPT-MET-DISPREV, Via Martiri di Montesole, 40129 Bologna, Italy; alessandro.gessi@enea.it; 2 GATEGREEN Srl, Via Armari 9, 44121 Ferrara, Italy; p.formaglio@gategreen.it (P.F.); b.semeraro@gategreen.it (B.S.); 3 Department of Chemical, Pharmaceutical and Agrarian Sciences, University of Ferrara, Via L. Borsari 46, 44121 Ferrara, Italy; daniela.summa@unife.it; 4 Department of Environmental and Prevention Sciences, University of Ferrara, Via L. Borsari 46, 44121 Ferrara, Italy; elena.tamisari@unife.it

**Keywords:** surface cleaning, sanitization, hypochlorous acid, HOCl, electrochemical technology

## Abstract

Recently, the use of disinfectants has been becoming a diffused and sometimes indiscriminate practice of paramount importance to limit the spreading of infections. The control of microbial contamination has now been concentrated on the use of traditional agents (i.e., hypochlorite, ozone). However, their prolonged use can cause potential treats, for both human health and environment. Currently, low-impact but effective biocides that are prepared in a way that avoids waste, with a very low toxicity, and safe and easy to handle and store are strongly needed. In this study, produced electrochemically activated hypochlorous (HOCl) acid solutions are investigated and proposed, integrated in a scrubbing machine for floor cleaning treatment. Such an innovative machine has been used for floor cleaning and sanitation in order to evaluate the microbial charge and organic dirt removal capacity of HOCl in comparison with a machine charged with traditional Ecolabel standard detergent. The potential damage on floor materials has also been investigated by means of Scanning Electron Microscope (SEM). A comparative Life Cycle Assessment (LCA) analysis has been carried out for evaluating the sustainability of the use of the HOCl-based and detergent-based machine.

## 1. Introduction

Nowadays, the use of disinfectants is a diffused practice and a fundamental precautionary action against the spread of infections and drug-resistant bacteria within hospitals, households and community settings [1]. Moreover, the current COVID-19 pandemic emergence is contributing to promote new hygiene protocols and procedures in healthcare environments as well as in the communities, schools, and social life in order to try to slow the spread of the virus, with the consequence of a huge increase in the use of antimicrobial hand sanitizers and disinfectant surface cleaners [2,3]. 

Significant benefits of chemical disinfection are the wide-availability, cost-effectiveness, and variety of applications on several surfaces without the requirement of mechanical devices [4]. A large number of chemical disinfectants, licensed by the US Environmental Protection Agency (EPA) or authorized by the Food and Drug Administration (FDA), are usually employed in hospital and household setting, including alcohols, sodium hypochlorite, formaldehyde, glutaraldehyde, hydrogen peroxide, ozone, peracetic acid, phenolics, and quaternary ammonium compounds [5]. 

The massive use of biocidal agents, originally limited almost exclusively for the healthcare sector or specific applications, has become over the last three years a widespread routine behavior for almost everyone and everywhere [6]. This means that it is necessary to assess the possibility that an indiscriminate application of disinfectants might compromise the in-use effectiveness in truly hygienic applications [7] and that could select for drug-resistant microbial strains or enhance the risk of cross resistance to antibiotics [8]. Moreover, other potential threats caused by the wider use of biocidal agents for disinfection could lead to the health risks or potential human or eco-toxicity derived from a prolonged exposure [9]. In fact, if overused or misused, disinfectants can also be dangerous to people and/or environment and can conduct to a neglected global crisis for health and ecosystems [10]. As recently reported by Samara et al. [11], several issues connected to intoxication have indicated how bleach products are responsible for the highest amount of poisoning during COVID-19 (62.1%), followed by non-alcohol disinfectants (36.7%) and hand-sanitizers (36.7%). They are principally inhaled by people contributing to poor indoor air quality with consequences for asthmatic, allergic, or sensitive people [12]. In fact, common surface disinfectants, such as the sodium hypochlorite recommended for use at a concentration of 0.1% or ethanol and isopropyl alcohol at 70% to 90% strength, are also recognized to induce eyes or skin irritation as well as to the respiratory tract [13]. Moreover, the use of sodium hypochlorite can lead to the formation of organochlorine compounds highly toxic for humans and environments [14]. It has been demonstrated that sodium hypochlorite persists in environment after 30 minutes since spraying, indicating that airborne droplets of hypochlorite can be a source of harmful exposure [15]. Alternatively, some hand sanitizers containing quaternary ammonium compounds, such as benzalkonium chloride, can provoke skin and respiratory system irritation and may induce asthma in some individuals. 

Ozone generators are usually encouraged as an efficient and eco-friendly option to sanity indoor air pollution and odours [16]. However, ozone is related with multiple harmful health consequences for years [17]. Scientific literature has highlighted that ozone concentrations that are secure to humans are doubtful to be active in controlling indoor air pollution, also provoking severe damages to materials, such as rubber, plastics, fabrics, paint, and metals [18]. A concentration of 0.3 ppm for 15 min has been indicated by the Occupational Safety and Health Administration (OSHA) as the human safety exposed-limit [19]. A recent study of Grignani et al. [20] reported that, at the same concentration, a virus 90%-inactivation time has been estimated in more than 100 min. 

A promising up-to-date disinfection technology based on nanomaterials has been proposed as a solution to overcome various constraints of chemical disinfectants, such as harmfulness, corrosive nature, and bacterial resistance, and have different sectors of application [21]. While the antimicrobial effect of silver and silver nanoparticles has been established, repeated exposure through various routes (skin, inhalation, or ingestion) to even safe doses could lead to health complications [22]. Moreover, nanosilver may well find its way into the environment via various routes, entering the food chain as well as the aquatic life [21].

Currently, green biocides that are active on microorganisms while provoking minimal or any damage to the environment, should also be prepared and packed to avoid waste, easy-to-handle and safely stored [23,24]. 

Electrolyzed water (EW) has been reported as a prospective substance that fulfils all the above requirements, together with low costs of production [25,26,27].

Different terms are reported to present water solutions that are produced by the electrolysis of salts, as for example electrochemically activated solutions (ECAS), electrolyzed oxidising water (EOW), or simply electrolyzed water (EW) [28]. The main species formed by the electrolysis of an aqueous NaCl solution is hypochlorous acid (HOCl), an unstable hydroxyl radical in a pH range from 2 to 7. HOCl has been indicated for years as a biocide agent for enhancing cutaneous wound healing and in the safe treatment of eye, nose, and ear infections [29,30,31,32,33,34]. Several studies have also demonstrated that HOCl is effective in water, air, and surfaces sanitization or disinfection [35,36,37,38,39]. Depending on the desired HOCl concentration, NaCl can be added to water, or it is also possible to use the amount present in groundwater and drinking water (usually <200 ppm) [26]. 

Microorganisms can colonize all surfaces, independent from the type of material and environmental conditions. Although some may be safe (i.e., *Lactobacilli*), others, due to their nature as human pathogens or their number, can be strongly harmful and have to be completely removed [40,41,42,43]. Surface adhesion or absorption mechanisms by microorganisms can be influenced by several factors, namely temperature, pH, relative humidity of the environment, and surface properties, such as porosity, composition, roughness, and hydrophilicity [4,44,45]. Among indoor surfaces, floors are the one of the most susceptible to microbial contamination due to the high probability of exposure to sources of contamination and dirt and to the presence of breakages that can retain moisture and nesting microbial colonies [46]. In this regard, studies carried out in hospitals, schools, and public spaces have shown that contaminated floors increase the probability to be infected not only by direct contact, but also by inhaling them as aerosols [47]. In fact, while walking on contaminated floors, microorganisms can be resuspended in air to man-height, increasing the microbial charge of indoor air up to 15% [48,49,50]. 

In this study, the HOCl electrochemical system has been integrated in a scrubbing machine for floor cleaning treatment. Such an innovative machine has been used for floors cleaning and sanitation in order to evaluate the microbial charge and organic dirt removal capacity of HOCl in comparison with a machine charged with traditional Ecolabel standard [51] detergent. A quartz-concrete floor has been tested in this study. Due to its durability and resistance, it is the most used floor materials for industrial floor, varying from food to heavy industries. Regardless, the surface damage potentially caused by prolonged HOCl applications on quartz-concrete has also been investigated by means of scanning electron microscope (SEM). Results have been compared with coated hardwood floor, usually considered more sensitive.

Furthermore, a comparative Life Cycle Assessment (LCA) analysis has been carried out in order to evaluate the environmental impact of cleaning operations using HOCl-based and detergent-based scrubbing machines. LCA is a standardized methodology (ISO 14040, ISO 14044, and ISO 14067) [52] for the validation of proper sustainability onward the overall production and use life cycle of a product, process, or service [52].

This study represents the first attempt to evaluate the overall effectiveness of HOCl solutions for floor cleaning treatments, including both the antimicrobial activities and the potential surface damages as well as the environmental impact based on LCA framework.

## 2. Materials and Methods

### 2.1. Experimental Area Description and Sampling Plan

**Figure 1:**
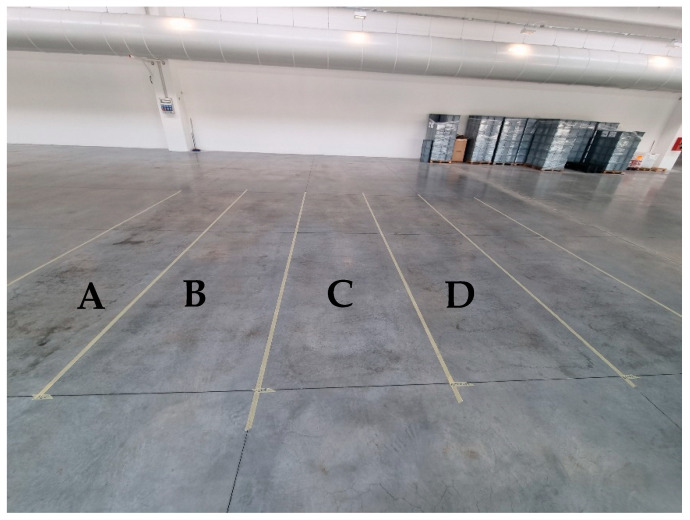
Test floor area marked out with strips: track A, as control, without any treatment; track B, with only water; track C, using Ecolabel^TM^ commercially available detergent; track D, with HOCl-based treatments.

The test floor area has been marked out with strips in 4 tracks of 1.5 m × 5.0 m each, previously cleaned and wiped (Figure 1). 

Floor area was made of quartz concrete. Scrubbing machine (MMG Plus, FIMAP, Italy) has a working width of 0.755 m and equipped with a washing brushes PPL 0.60 with plastic disc diameter 400 mm. Operating parameters of the scrubbing machine used in these experiments have been reported in Table 1.

### 2.2. Scrubbing Machine Integrated with HOCl Production System

**Figure 2:**
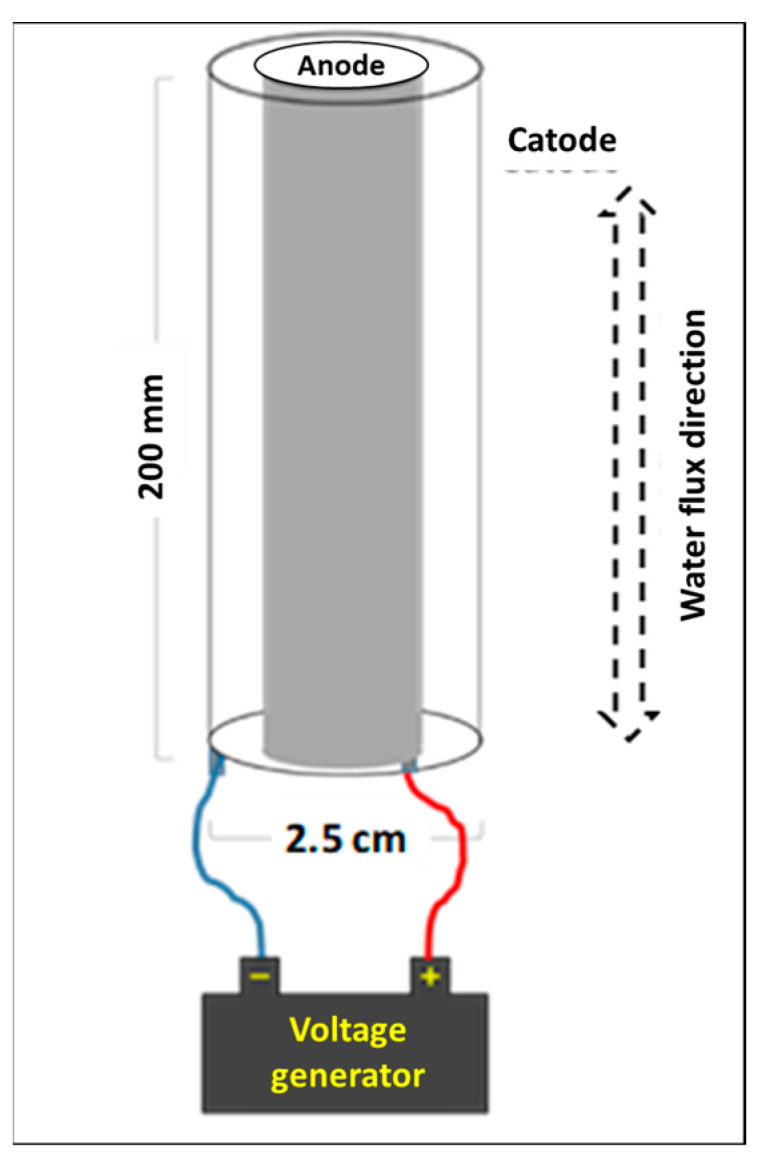
Scheme of the electrochemical cell for HOCl production from tap water.

The scrubbing machine was integrated with 2 electrochemical cells for the in situ production of HOCl solution. Each electrochemical cell had a flow-through tubular shape (Figure 2), consisting of cylindrical coaxial parts. An internal anodic rod (external diameters 8 mm, length 173 mm) made of titanium, provided with an iridium metal oxide-based catalytic coating and an external cathodic titanium cylinder (internal diameter 14 mm, length 232 mm), were used.

HOCl in aqueous solution is produced by electrochemical reaction generated by a voltage of 24 V. In the presence of NaCl, usually present in tap water, in Italy, at a concentration 25–30 ppm, oxygen (O_2_) and chlorine (Cl_2_) are synthesized at the anode of the electrochemical reactor, both in gaseous form. Once formed, the two gases follow different paths. As oxygen moves away from the reaction environment, chlorine is able to dissolve in water, producing HOCl. HOCl, in aqueous solution, is always in equilibrium with the corresponding hypochlorite ion, ClO^−^.

HOCl (aq) + H_2_O (l) <-> OCl^−^ (aq) + H_3_O^+^ (aq) (1)

**Figure 3:**
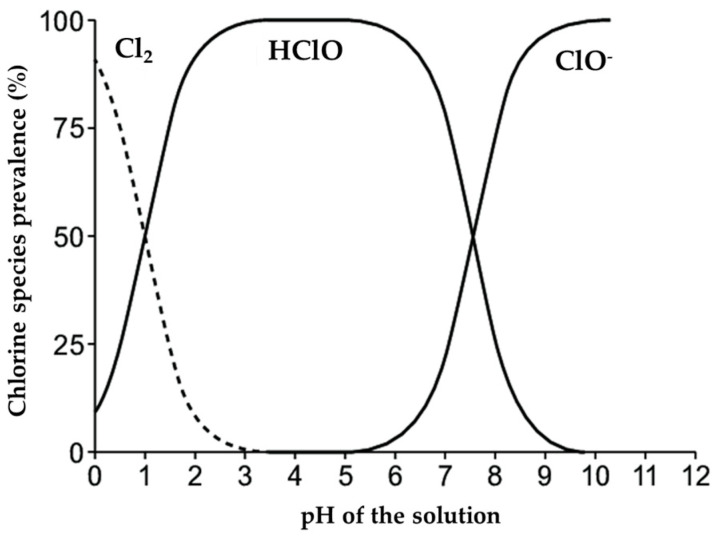
The relative percentages of three different chlorine species in solution as a function of pH [54].

The pH of the solution determines that forms from chlorine will be present (HOCl /OCl^−^/Cl_2_) and represents the key factor to explain the sanitizing effectiveness of the solution coming out of the electrochemical cell. If the pH of the solution is kept around the value 6–7, it is guaranteed the presence of >95% HOCl, which is, among the 3 possible forms of chlorine, the most oxidizing and therefore most effective as a sanitizer (Figure 3) [53].

At an average 25–30 ppm chloride concentration and slightly-acidic pH (pH = 6.5), in tap water, the electrochemical cells yield of conversion was about 30%, obtaining a final HOCl concentration of 10 ppm. HOCl concentration was checked by means of the photometric DPD (N,N-diethyl-p-phenylenediamine) assay [55]. 

### 2.3. Cleaning Test: Removal of Organic Dirt from Surface

The test strips was divided in six sub-areas of 0.42 m^2^ each, marked with colored tape strips. Each sub-area was nebulized with 15 mL of a protein and lipid-based standard solution and left to dry naturally. After 24 h, cleaning conditions, as organic dirt removal capacity, were measured in three sub-areas using qualitative clean test kit [56]. The clean test consisted of a buffer for the detection of organic residues (proteins, fats, and sugars) specifically designed to check the level of cleanliness of a given work surface. The test provided qualitative data based on photometric indications. The product was composed of test tubes plastic test tubes containing a buffer, a vial (reagent A: bicinchoninic acid-BCA), and a floppy disc reagent disc (reagent B: CuSO_4_)).

### 2.4. Sanitization Test: Removal of Microbial Charge from Surface

Using the procedure previously described at 2.3, the remaining three sub-areas were sampled using RODAC (Replicate Organism Detection and Counting) contact Petri plates (Liofilchem, Teramo, Italy). Adhered microorganisms were transferred directly to the plates via direct contact under standardized conditions (applying 0.02 kg/cm^2^ of constant pressure for 10 s). The plates had a surface area of 24 cm^2^ and a bottom grid to facilitate the counting of colonies. Mesophilic bacteria and fungi were detected. Selected cultivation media were used for bacteria (Plate Count Agar at pH = 7.0 ± 0.2), fungi (Sabouraud Cloramfenicol Agar at pH = 5.6 ± 0.2) [57]. Mesophilic bacteria and fungi were incubated at 37 °C for 48 h and 22 °C for 120 h, respectively.

RODAC plates were then incubated within 2 h, colonies were counted after 24–48 h. All sampling was carried out in triplicate.

A total of 144 samples were collected. Samples were transported to the laboratory in refrigerated insulated bags (4 °C). Microbial density was expressed in terms of CFUs (Colony-Forming Units)/100 cm^2^ and calculated as:

R = Ut − At(2)

where:

R = microbial charge reduction;

Ut = average value of the number of colonies recovered on control surface (track D) after 24 h, expressed as Log10;

At = average value of the number of colonies recovered on treated surface (tracks A, B or C) after 24 h, expressed as Log10.

Finally, R% was derived from the following formula:

R% = (1 − 10^−R^) × 100 (3)

The microbial density, based on INAIL (Istituto Nazionale Assicurazione Infortuni sul Lavoro—National institute for insurance against industrial injuries) standard protocols for civil environments, should be also expressed in terms of CFU/100 cm^2^, calculated by dividing the count result (N, number of colonies/plate) by the contact area of the plate with the test surface (24 cm^2^):

CFU/100 cm^2^ = N/24 × 100 (4)

A very slight growth (<40 colonies/100 cm^2^ ≅ 10 CFU/24 cm^2^) was considered as acceptable for the standard [58].

### 2.5. Surface Damage Evaluation with SEM

The Scanning Electron Microscope (SEM Quanta Inspect S, FEI Company, Eindhoven, The Netherlands), 5 nanometers resolution and equipped with an EDS (Energy Dispersive System) probe (Oxford Instruments, Abingdon, UK), was used. A small sample of quartz concrete (10 mm × 10 mm) and coated hardwood floor (10 mm × 10 mm) were softly cleaned in order to remove any layers or deposits of dust. Samples were then sprayed with a concentrated HOCl solution at 2000 ppm, appropriately created adding NaCl to water. After exposure, the samples were wiped in order to simulate the scrubbing operation. For observation under the SEM microscope, samples were placed on conventional aluminum stubs, made conductive with a carbon adhesive disc. In order to gather significant elemental mapping, the SEM parameters were set to 30 Kv and spot 7, allowing for a faster and more accurate EDS acquisition. 

### 2.6. LCA Methodology

In this study, LCA followed the standardized method, regulated by ISO 14040 series, which consists of four stages: (1) goal and scope definition; (2) life cycle inventory construction; (3) environmental impact assessment; and (4) interpretation of the results.

LCA was carried out using OpenLCA™ software (Green Delta, Berlin, Germany) and applying the CML Baseline 4.4 method. The goal and scope was the quantification of the environmental impact of a scrubbing machine used for cleaning service in civil buildings in Italy, equipped with HOCl production system in comparison with a traditional detergent-based machine. According with the 14,040: 2006 (Environmental management—Life Cycle Assessment—Principles and frame work), the functional unit (FU) is 1 m^2^ of cleaned surface by a scrubbing machine. Data collection for life cycle inventories included specific data collected directly during the cleaning tests, whereas all background data on the production of electricity (i.e., *electric energy mix, at grid-IT*) and tap water (i.e., *tap water production, conventional treatment, at user-EU*) as well as secondary data on raw materials production and transportation have been derived from the Ecoinvent™ 3.8 database. All the data used refer to 2023, the year in which the service was provided. The system boundaries include:

(1) Upstream processes: raw materials production for machine construction and packaging;
(2) Core processes: supply chain and transportation of raw materials, production of electricity, and water consumption for semi-finished and finished machine assembling;
(3) Downstream processes: machine use and maintenance, transportation to the experimental site, waste management, and machine end-of-life.

For the present study, it has been assumed that the following characteristics were identical in the compared systems and consequently excluded by the boundaries:

- the transport of cleaning operators involved, technical vests, and any PPE used during cleaning;
- the building and the area for machines construction;
- data quality.

Based on the available data, the output of the analysis was that the environmental impact has been evaluated by the quantification of the mid-point impact categories (Table 2). 

ODP potential accounts for impacts related to the reduction of the protective ozone layer within the stratosphere caused by emissions of ozone depleting substances, with the final impact indicator indicating mass (e.g., kg) of equivalent CFC-11. POCP measures the contribution of ethylene and other volatile organic carbon compounds in the presence of heat and sunlight to ozone formation at ground level. The reference substance is ethylene. ADP refers to the removal of abiotic resources from the earth or the depletion of non-living natural resources, and antimony is considered as reference because of its wide distribution and scarcity on earth. HTP and ETP reflect the potential harm of a unit of chemical released into the environment based on both the inherent toxicity of a compound and its potential dose. The 1,4-dichlorobenzene (1,4-DCB) is the reference for toxicity indicator. GWP evaluates the contribution at a time-horizon of 100 years to global warming of a substance that is released into the atmosphere, measured as unit mass of CO_2_. AP measures the amount of sulfur dioxide (SO_2_) and oxides of nitrogen emissions in atmosphere derived from combustion that react with water vapour and form acids, which fall down as acid rain or snow. EP quantifies the nutrient enrichment in water by the release of nitrogen and phosphorous, which provokes the abnormal growth of algae. Sulfur dioxide and phosphate were the corresponding reference substances for AP and EP, respectively.

## 3. Results

### 3.1. Cleaning Effectiveness

**Figure 4:**
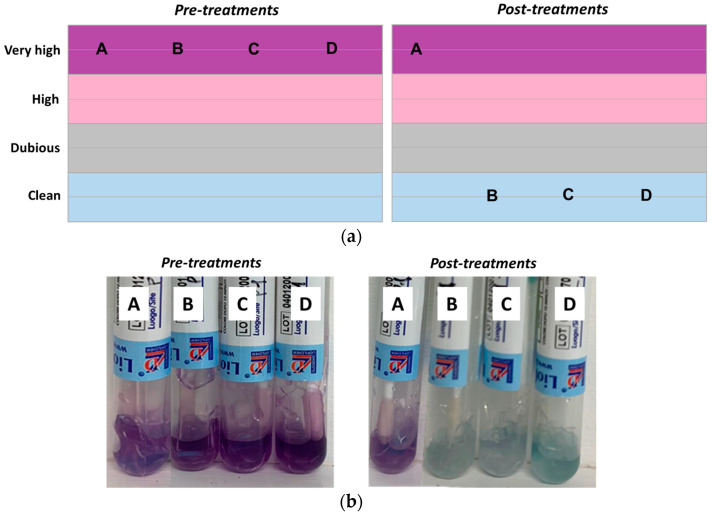
Qualitative depiction of CLEANING TEST results (**a**) and test tubes results (**b**). Depending on the level of organic dirt, the test solution colored from violet to light blue. track A, as control, without any treatment; track B, with only water; track C, using Ecolabel^TM^ commercially available detergent; track D, with HOCl-based treatments.

The effect of organic dirt removal due to treatments applied to surface tracks have been reported in Figure 4.

Before the treatments, floor surfaces appear very dirty, whereas in the post-treatments, B, C, and D sub-areas showed the complete removal of organic residues on surfaces differently from the untreated track (A), as expected.

### 3.2. Microbial Charge Reduction Effectiveness

**Figure 5:**
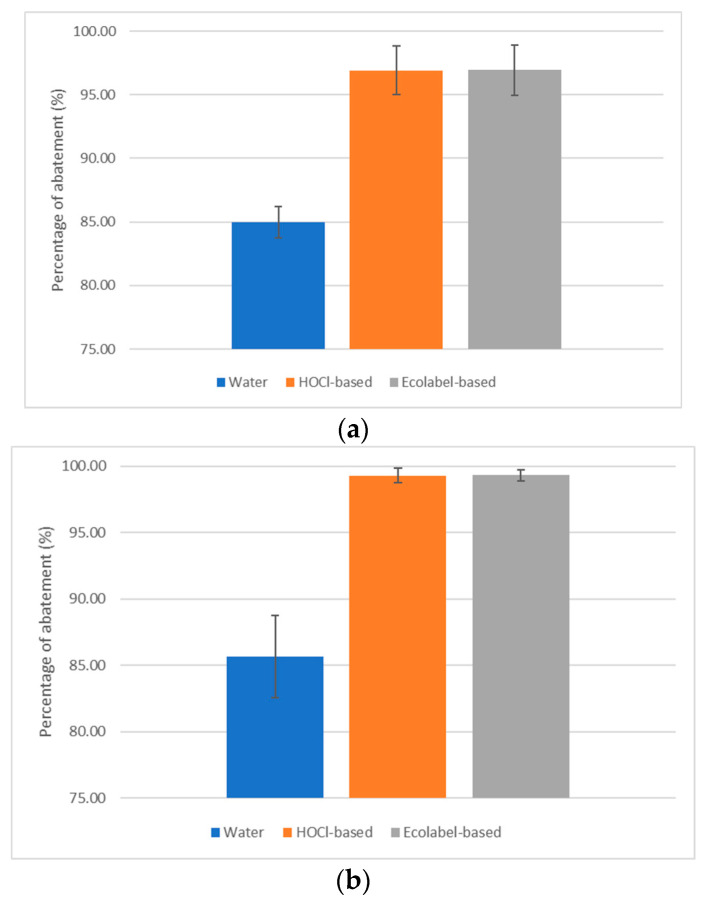
Percentage of abatement of bacterial (**a**) and fungal (**b**) charges on the three selected tracks areas (blue bar, water; orange bar, HOCl-based; grey bar, Ecolabel-based detergent) between pre-treatment and post-treatment. Data are the average of 2 independent experiments performed in triplicate (average ± standard deviation), and values are represented as a percentage of reduction.

The comparison among the different sanitization protocols (i.e., with water, HOCl and detergent) has evidenced that, except for cleaning with only water, the percentage of abatement of microbial charge were found acceptable (Figure 5).

Percentage reduction of bacterial counts was 84.9 ± 1.2%, 96.9 ± 1.9%, and 96.9 ± 2.0% for water, HOCl-based, and detergent-based treatment, respectively. Percentage reduction of fungal counts is 85.6 ± 3.1%, 99.3 ± 0.5%, and 99.3 ± 0.4% for water, HOCl-based, and detergent-based treatment, respectively.

Treatment based on the use of only water is significantly less effective than treatments based on the use of additives to water. The percentage of abatement by means of water alone is due to the effect of mechanical removal.

After treatment with both HOCl and detergent, the number of CFU/100 cm^2^ was <40 bacterial colonies (37.3 CFU/100 cm^2^ and 39.1 CFU/100 cm^2^ respectively), as is requested to consider acceptable for microbial removal, whereas the treatment with water alone do not warrant the correct sanitization, with a residual contamination of about 306.2 CFU/100 cm^2^.

### 3.3. SEM Observation

**Figure 6:**
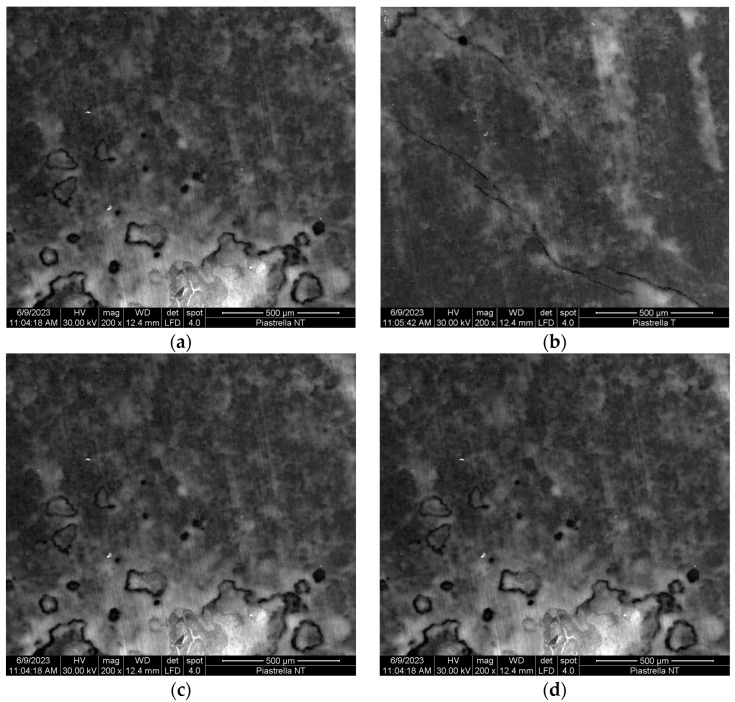
Effects of 2000 ppm HOCl treatment on floor surfaces analyzed by SEM-EDS microscopies; (**a**) SEM image of quartz concrete untreated; (**b**) SEM image of quartz concrete HOCl, 2000 ppm treatment; (**c**) SEM image of coated hardwood floor untreated; (**d**) SEM image of coated hardwood floor HOCl, 2000 ppm treatment. No salt crystals are apparent.

To clarify the effect of HOCl treatment on floor surface and, consequently, to evaluate the potential damages, the surface morphologies were observed using SEM before and after the treatments. Samples were over-exposed to HOCl solutions, i.e., at HOCl-2000 ppm, in order to test floors in the strongest possible conditions. Potential damages due to HOCl treatment have been tested on quartz concrete and on coated wooden floor as reference materials. No apparent change in the surfaces’ morphology as well as no saline crystal deposition evidence has been found (Figure 6). 

**Figure 7:**
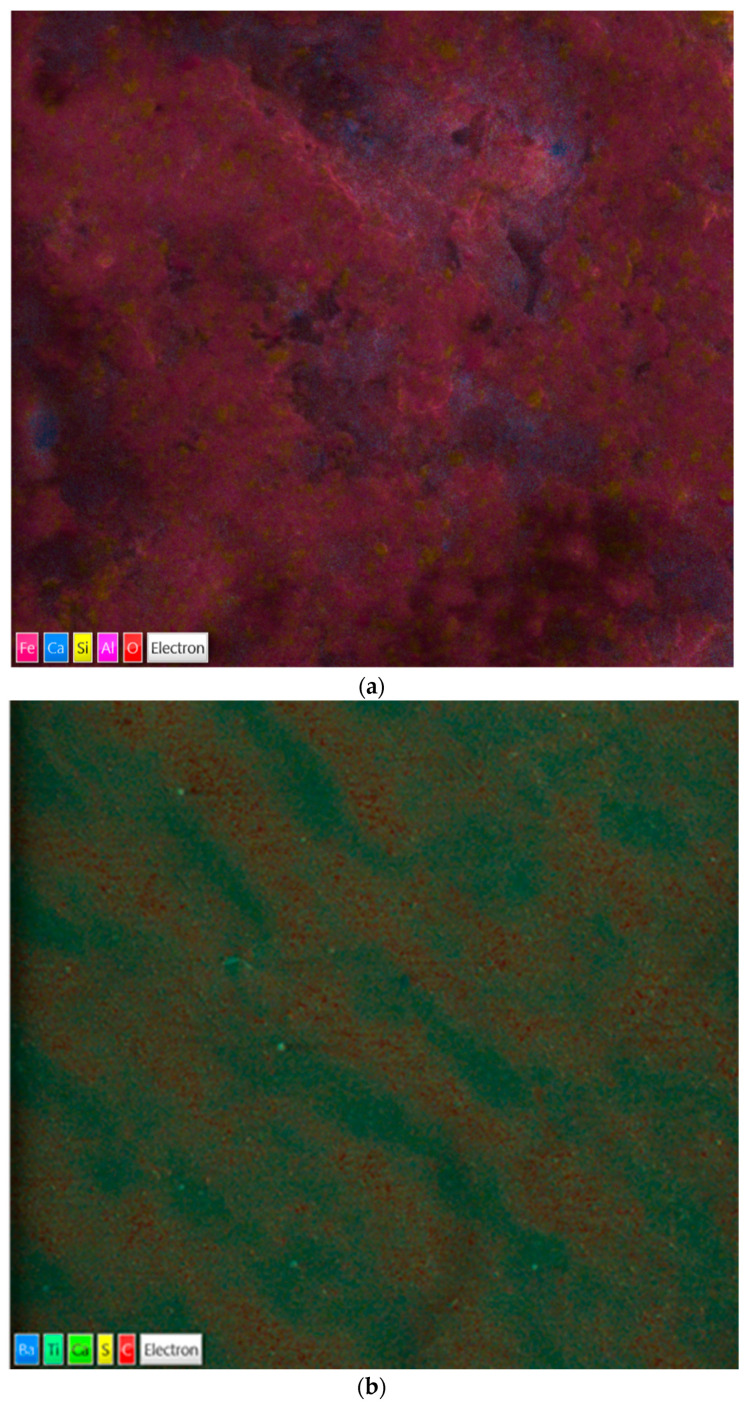
Effects of 2000 ppm HOCl treatment on floor surfaces analyzed by SEM-EDS microscopies: EDS mapping; (**a**) MAP on treated quartz; (**b**) Map on coated hardwood floor, 2000 ppm treatment. Artificial colors correspond to the various element present in the surface.

In Figure 7, the overlayed elemental maps of the two surfaces analyzed are reported. Where standard micrographies give morphological information, i.e., possible surface modifications, corrosion, and scale deposition, the overlayed colour maps describe the relative distribution of the detected elements by means of the EDS probe. 

In this specific experiment, micrographies could have revealed a saline crystal deposition, and EDS maps could have described Na and Cl distribution over the surface. Any single element is artificially depicted with a different colour. Otherwise, surface maps permit to clearly distinguish the predominant presence of xygen (red) and silica (yellow) of quartz, aluminum (dark pink), and calcium (blue) of concrete (Figure 6a) and the presence of titanium (dark green) of hardwood varnish and carbon (red) of wood. 

Semi-quantitative microanalysis data from SEM-EDS show almost identical elemental composition for both materials, before and after the treatments with HOCl. Elemental mapping (Figure 6a,b) after HOCl exposure represent with artificial colours the relative concentration of the surface elements. There is no detectable Na or Cl presence. In Table 3, the data clearly spot no difference in samples regarding NaCl residual deposition as well as the presence of traces of oxidation. All the floor samples have shown no damage effects due to HOCl. 

High presence of oxygen (O) is due to the fact that metals are usually in the form of inorganic oxides. In particular, quartz is a crystalline mineral composed of silica dioxide. Concrete is composed of silica (Si), iron (Fe), aluminium (Al), and calcium (Ca) oxides, whereas titanium (Ti) and barium (Ba) are used as common elements in wood paints. In fact, parquet floors are never rough wood made but nitrocellulose or polyurethane painted. Metals are commonly part of such kind of paints.

It must be underlined that possible salt depositions are normal consequences of the HOCl application method, but the effect is strongly dependent on surface roughness. Both coated hardwood floor and quarts-concrete are characterized by a smooth surface, allowing for a very effective mechanical cleaning of the surfaces.

### 3.4. LCA Analysis

For LCA results, a 10^−3^ cut-off has been applied. Values below the cut-off have been considered to give a negligible contribution to the overall environmental impact (Table 4).

The numerical values of other impact categories were below the applied cut-off and so not included in the analysis. For all reported categories, the HOCl-based system has shown lower environmental impact. The *cradle-to-grave* analysis carried out on the two scrubbing systems has evidenced that the use of HOCl-based machine leads to an overall GWP reduction of about 30% per square meter. The higher values corresponding to the detergent-based system is mainly due to the use and disposal of residual water after surface washing and drying. The absence of added chemicals and the opportunity of disposing the residual water as tap water reflect significant environmental benefits, not only in terms of GWP emissions, but also in terms of HTP and ETP.

Focusing on GWP, the contribution of different processes and phases is illustrated in Table 5. 

The higher impact of the detergent-based system is related to the use of detergent, corresponding to about 35% of the overall impact of detergent-based scrubbing machine, whereas HOCl-based machines generate higher emissions in the upstream phase due to the increased weight of the electrochemical cells. In particular, the upstream processes inventory has shown that the two machines have been built with identical components, except for the two-electrochemical cells unit in the HOCl-based machine, resulting in a higher weight (218.67 kg vs. 214.18 kg). Moreover, electricity consumption during the machine use leads to higher GWP value. In the detergent-based machine the use and disposal of detergent account for 57% of the overall GWP. Water consumption, machine maintenance, and end-of-life do not significantly contribute to the GWP impact.

## 4. Discussion

The use of chemically based detergents and disinfectant agents has rapidly increased in the last year, especially since the COVID-19 pandemic, despite their potential toxicity to humans and the environment. In addition, the habit of disinfection has become part of the cleaning practice, even in sectors where cleaning has traditionally been seen as the removal of inorganic and organic dirt rather than the removal of microorganisms. 

In this study, the cleaning and sanitizing performance of an innovative system based on HOCl and a traditional eco-labelled detergent have been compared. HOCl has the advantage of leaving no chemical residues on the surfaces or in the residual water because once it has oxidized the dirt and microorganisms, it is deactivated and reverts to NaCl. In the present application, a HOCl concentration of 10 ppm was achieved using only the concentration of NaCl normally present in tap water, without the need of an external salt solution supply. The scrubbing machine equipped with electrochemical cells is capable of cleaning and sanitizing the floor surface by means of tap water only, properly activated. The results have shown no significant differences in both organic dirt and microbial removal between the Ecolabel detergent and HOCl-based systems. The two treated tracks (C and D) were found acceptably cleaned and sanitized, and the effectiveness of the HOCl-based system has always been comparable to the Ecolabel detergent.

Moreover, the HOCl-based solution has been shown not to damage floor surfaces even after more than a thousand applications and to be safe also for sensitive surfaces used as the control. Unlike bleach or chlorine solutions, HOCl is non-corrosive on surfaces or equipment because of slightly acidic pH conditions. The optimal pH range with >95% of HOCl in solution was between 5.5 and 6.5. Theoretically, at this interval, HOCl is the predominant chlorine form. In contrast, alkaline conditions (pH > 8.0) cause hypochlorite ions to predominate, and a very low pH favors the formation of chlorine gas, both toxic and corrosive [59]. Moreover, extensive research has confirmed that HOCl is the most effective antimicrobial form of chlorine [60]. 

Quartz concrete is the most used floor material in both commercial and recreational spaces and has to be tough and durable. SEM-EDS images have shown that HOCl does not damage the surface floor at all, even after a number of applications. The specific *wash and dry* cleaning method operated by the machine also avoids NaCl deposition on floor surface.

The comparative LCA analysis showed that the use of the HOCl system integrated in a scrubbing machine for floor cleaning and sanitization permits avoiding about 30% of emissions per square meter compared to the use of the Ecolabel detergent. The environmental value added from the HOCl-based machine is even more clear if the scenario of application on an annual basis is considered. Considering a machine productivity of 2250 m^2^ per hour worked (see Table 1) and an average annual use of about 250 h per year, the cleaning service provided with the HOCl-based machine leads to a reduction of 230 kgCO_2_ eq./year for a single machine. Even with the higher emissions in the upstream processes due to the inclusion of electrochemical cells and higher electricity consumption in the use phase, the complete elimination of the detergent determines a significant reduction in the environmental impact.

## 5. Conclusions

At a time when there is the urgent need for cleaning and sanitizing as a consequence of the pandemic, a system that ensures a reduction in environmental impact is paramount in the management of cleaning civil environments. This work provides important insight into HOCl-solution application during industrial cleaning practices. The opportunity to combine the effectiveness of cleaning and absence of chemical residuals with lower emissions could represent an innovative approach. Moreover, surfaces are not damaged at all by the prolonged use of HOCl solutions. It is worthwhile noting that a satisfying level of organic dirt removal and antimicrobial decontamination has been reached, but it also saves about 30% of CO_2_ emissions per square meter and avoids the use of chemical detergents. 

The HOCl solution is already in use in many industries from farming and restaurants, regarding food, to health care applications, and floor cleaning and sanitation may represent an additional field of development of this technology. In fact, HOCl comprises many of the desired effects of the ideal disinfectant: it is easy to use, is inexpensive, has a good safety profile, and can be used to disinfect large areas quickly and with a broad range of cidal effects. 

## Figures and Tables

**Table 1:** Operating parameters of the scrubbing machine used for the experimental tests.

Parameter	Machine Setting
Washing brushes push (kg)	70
Speed rate (km/h)	3
Flow rate (L/min)	0.9
Working capacity (m^2^/h)	2250
EW consumption (L/100 m^2^)	2.4
Specific pressure (g/cm^2^)	37

**Table 2:** Impact categories considered in this study as output of LCA analysis. Unit is expressed as kilograms of reference substance equivalent.

Impact Categories	Unit
Ozone depletion potential (ODP)	kg CFC-11 eq.
Photochemical ozone creation potential (POCP)	kg ethylene eq.
Abiotic resources depletion potential (ARDP)	kg Sb eq.
Human toxicity potential (HTP)	kg 1,4-DCB eq.
Global warming potential (GWP)	kg CO_2_ eq.
Ecotoxicity potential (ETP)	kg 1,4-DCB eq.
Acidification potential (AP)	kg SO_2_ eq.
Eutrophication potential (EP)	kg PO_4_^3−^ eq.

**Table 3:** Semi-quantitative microanalysis data from SEM-EDS spectrum for the two floor materials (quartz concrete and hardwood) before and after HOCl treatments.

	Quartz Concrete		Ardwood Floor	
Element	Untreated	Treated	Untreated	Treated
C	17.84 ± 0.19	15.81 ± 0.16	56.70 ± 0.08	56.42 ± 0.07
O	43.51 ± 0.11	43.92 ± 0.10	26.61 ± 0.07	26.56 ± 0.06
Na	0.70 ± 0.01	0.77 ± 0.01	-	-
Mg	1.63 ± 0.01	1.63 ± 0.01	0.04 ± 0.00	0.04 ± 0.00
Al	6.19 ± 0.02	6.61 ± 0.02	0.61 ± 0.00	0.62 ± 0.00
Si	17.76 ± 0.05	19.13 ± 0.04	1.15 ± 0.00	1.16 ± 0.00
P	0.87 ± 0.01	0.77 ± 0.01	-	
S	0.25 ± 0.00	0.28 ± 0.00	1.40 ± 0.00	1.42 ± 0.00
Cl	0.06 ± 0.00	0.06 ± 0.00	0.06 ± 0.00	0.06 ± 0.00
K	1.85 ± 0.01	1.93 ± 0.01	0.07 ± 0.00	0.07 ± 0.00
Ca	4.55 ± 0.01	4.22 ± 0.01	2.34 ± 0.01	2.43 ± 0.01
Ti	0.37 ± 0.00	0.39 ± 0.00	4.11 ± 0.01	4.24 ± 0.01
Mn	0.12 ± 0.00	0.12 ± 0.00	-	-
Fe	4.04 ± 0.01	4.08 ± 0.01	-	-
Zn	0.18 ± 0.01	0.15 ± 0.01	-	-
Sr	0.07 ± 0.02	0.07 ± 0.02	0.11 ± 0.01	0.11 ± 0.01
Ba	-	-	6.80 ± 0.02	6.92 ± 0.02
Total	100	100	100	100

**Table 4:** Impact categories related to the main processes and phases of life cycle related to the two scrubbing systems compared in this study.

Impact Categories	HOCl-Based Scrubbing Machine	Detergent-Based Scrubbing Machine	Unit
HTP	0.19	0.12	g 1,4-DCB eq./1 m^2^
GWP	1.37	0.96	g CO_2_ eq./1 m^2^
ETP	0.068	0.053	g 1,4-DCB eq./1 m^2^

**Table 5:** GWP related to the main processes and phases of life cycle of the two scrubbing systems compared in the present study.

Process	Phase	HOCl-Based Scrubbing Machine (gCO_2_ eq./m^2^)	Detergent-Based Scrubbing Machine (gCO_2_ eq./m^2^)
**Upstream**	Raw materials production	0.42	0.34
**Core**	Supply chain and transportation of raw materials	0.03	0.03
	Electricity and water consumption for machine assembling	0.02	0.02
**Downstream**	Use—energy consumption	0.37	0.31
	Use—water consumption	0.01	0.01
	Use—detergent consumption	0.00	0.48
	Transportation to the experimental area	0.00	0.00
	Machine maintenance	0.08	0.08
	Machine end-of-life	0.01	0.01
	Waste management	0.03	0.09
	**TOTAL**	**0.96**	**1.37**

## Data Availability

Not applicable.
